# Hematopoietic stem cell transplantation and immunosuppressive therapy: implications of clonal haematopoiesis

**DOI:** 10.1007/s00277-024-06152-6

**Published:** 2025-01-28

**Authors:** Zhengwei Tan, Xinhe Zhang, Jia Feng, Yuechao Zhao, Huijin Hu, Dijiong Wu, Qinghong Yu, Yu Zhang, Liqiang Wu, Tonglin Hu, Zhengsong Yan, Baodong Ye, Wenbin Liu

**Affiliations:** 1 Department of Hematology, The First Affiliated Hospital of Zhejiang Chinese Medical University (Zhejiang Provincial Hospital of Traditional Chinese Medicine), Hangzhou, China; 2 The First School of Clinical Medicine, Zhejiang Chinese Medical University, Hangzhou, China

**Keywords:** Aplastic anemia, Hematopoietic stem cell transplantation, Immunosuppressive therapy, Clonal hematopoiesis, Somatic mutation

## Abstract

Aplastic anemia (AA) is a life-threatening bone marrow failure syndrome. The advent of next-generation sequencing (NGS) has shed light on the link between somatic mutations (SM) and the efficacy of immunosuppressive therapy (IST) in AA patients. However, the relationship between SM and hematopoietic stem cell transplantation (HSCT) has not been extensively explored. In this retrospective analysis, we examined 166 AA patients who received HSCT or IST at our institution between May 2019 and December 2023. NGS was conducted on 66 genes within bone marrow cells to investigate the correlation between SM and the prognosis and therapeutic response in AA patients, as well as to assess the impact of mutation types on HSCT outcomes. Clinical data were gathered from 166 AA patients, comprising 84 males and 82 females, with a median age of 32 years (ranging from 9 to 75 years). In our study, a total of 151 somatic mutations were identified across 84 patients (50.6%), with 42 patients (25.3%) presenting a single mutation and 26 patients (15.7%) harboring two mutations. The top five genes with the highest mutation frequency were BCOR/BCORL1 (12.6%), ASXL1 (8.6%), TET2 (6.6%), CEBPA (5.3%), and GATA2 (4.6%). We stratified patients into SM and No-SM groups based on the presence of mutations and further divided them into HSCT and IST groups to assess the influence of mutation types on treatment response and survival within and between these groups. The findings were as follows: 1.Patients in the HSCT group exhibited a higher treatment response (OR 85.9% vs. 68.4%, *p* < 0.05), although there was no significant difference in survival. 2.Patients with favorable mutations, such as PIGA and BCOR/BCORL1, experienced significantly improved response and survival compared to those with unfavorable mutations like ASXL1, DNMT3A, and TET2 (OR 93.7% vs. 72%, *p* < 0.05) (3-year OS 93.7% vs. 80%, *p* > 0.05). 3.The HSCT-Favorable group demonstrated superior response rates (OR 100% vs. 67.7%, *p* < 0.05) and longer survival (3-year OS 100% vs. 67.7%, *p* < 0.05) compared to the IST-Favorable group. This study underscores that AA patients carrying favorable mutations, particularly BCOR/BCORL1, tend to have a more robust response and better prognosis than those without mutations or those with unfavorable mutations, such as ASXL1/DNMT3A. These findings are especially pertinent to HSCT, highlighting the importance of NGS prior to initiating treatment.

**Supplementary Information:**

The online version contains supplementary material available at 10.1007/s00277-024-06152-6.

## Introduction

Aplastic anemia (AA) is an acquired bone marrow failure disease characterized by low bone marrow hematopoietic function and pancytopenia [1]. Currently, Immunosuppressive therapy (IST) and Hematopoietic stem cell transplantation (HSCT) are the mainstays of AA treatment [2, 3]. Allogeneic hematopoietic stem cell transplantation (Allo-HSCT) is the preferred treatment for young AA patients with HLA-matched related donor (MRD), whereas IST is preferred for patients without MRD and aged > 40 years [4, 5], and HSCT can also be considered as an alternative choice for patients without MRD or those who fail initial IST [6].

The development of next-generation sequencing (NGS) technology in recent years has provided the basis for the diagnosis and treatment in various of hematological malignancies, and an increasing number of studies have revealed the regularity of Clonal hematopoiesis (CH) and the correlation between SM and the response and survival of IST in AA patients. For example, Park and Yoshizato [7–9] showed that the most common mutations in AA patients were BCORL1/BCOR, ASXL1, DNMT3A and TET2, among which AA patients with myeloid tumor-related mutations, such as ASXL1, DNMT3A, RUNX1, showed approximately 40-60% response and overall survival after IST. Some studies have even shown that the risk of coronary artery disease in carriers of DNMT3A, TET2, ASXL1 has nearly doubled, and the risk of myocardial infarction is 4-fold greater than noncarriers [10–12], whereas AA patients carrying mutations, such as BCOR, BCORL1 and PIGA, tend to have a better response and improved overall survival. However, there are few studies on how CH affects the response and survival of HSCT. The purpose of this study is to investigate regularity of CH in AA patients, and the correlation between the types of SM and the response and survival, so as to guide the treatment of patients with different types of mutations.

## Patients and methods

### Patients

This study was reviewed and approved by the Ethics Committee of The First Affiliated Hospital of Zhejiang Chinese Medical University, and all patients and donors signed written informed consent form before receiving treatment. This study follows the guidelines of the Declaration of Helsinki. The diagnosis of AA was in accordance with the Camitta criteria [6]. From May 2019 to December 2023, a total of 166 eligible AA patients were included in this study.

The inclusion criteria: (1) Consultation from May 2019 to December 2023; (2) HSCT or IST treatment; (3) NGS; (4) Complete clinical data.

### Treatment regimen

HSCT: All patients were admitted to the laminar flow ward from the start of treatment; donor selection included haploidentical, sibling allogeneic, and unrelated donors; conditioning regimens included FCA regimen (Fu 30mg/m^2^/d,−9d to−5d; CTX 20–40 mg/kg/d,−5d to−2d; ATG, Sanofi, rabbit source, 2.5-3.5 mg/kg/d−5d to−2d), Bucy regimen (Bu 3.2/kg/d,−7d to−6d; CTX 50 mg/kg/d,−5d to−2d; ATG, Sanofi, rabbit source, 2.5 mg/kg/d−5d to−2d), FABT regimen (ATG, Sanofi, rabbit source, 2 mg/kg/d, -7d to -5d; Flu 30mg/m^2^, -7d to -3d; Bu 3.2 mg/kg, -4d; CTX 25 mg/kg/d, -3d to -2d; TT 5 mg/kg,-2d), and use MMF and CsA (or FK506) to prevent graft rejection and GVHD after HSCT, respectively. The distinction lies in the fact that the FABT group received high-dose CTX, whereas the FCA and Bucy groups were treated with a short course of methotrexate (MTX). Specifically: +3d to + 4d, CTX 40 mg/kg/d. Or + 1d, MTX 15mg/m^2^; +3d, +6d, + 11d, MTX 10 mg/m^2^.

IST: All patients were admitted to the laminar flow ward from the start of treatment; patients in the IST group were treated with ATG + CsA; ATG (Sanofi, rabbit source, 3 mg/(kg·d), 5d) + CsA (5 mg/(kg·d), administered for at least 1 years, maintaining the trough concentration at 200-250ng/ml), routine prophylactic anti-infective, antifungal and antiviral therapy was performed during immunosuppression.

### Definition of response

Efficacy grading according to NIH criteria [13].

Complete response (CR): HGB > 100 g/l, PLT > 100 × 10^9^/l, ANC > 1.5 × 10^9^/l, not dependent on blood transfusion;

Partial response (PR): withdrawal from component blood transfusion (HGB > 70 g/l, PLT > 20 × 10^9^/l), improvement of hematological indices, no longer fulfilling SAA diagnostic criteria;

No response (NR): Patients who were continuously transfusion-dependent and/or still fulfilled the diagnostic criteria for SAA and who died from various causes after HSCT or IST treatment;

Overall response (OR):CR + PR.

### Gene sequencing

NGS was used to analyze the gene mutation of DNA extracted from mononuclear cells in patients' bone marrow samples. A library with an inserted fragment of 200–250 bp was constructed. The target sequence was captured by high-performance liquid phase probe. The Illumina Next500 sequencing platform was used to perform 2 × 150 bp Paired-end (PE) sequencing on the library. The detection sensitivity was 1%, that is, genes with a variant allele frequency (VAF) greater than 1% could be detected. According to the VAF diagnostic threshold for CH established by the team of Jaiswal S [14, 15], the inclusion threshold for SM in this study was defined as 2%.

In this study, NGS was used to detect 66 AA-associated SM including ABL1, ASXL1, ATM, etc. The detection range included hot spot mutations (SNV and Indel) and FLT3-ITD in the gene coding region. (The specific mutant genes are shown in Table 1).

**Table 1:**
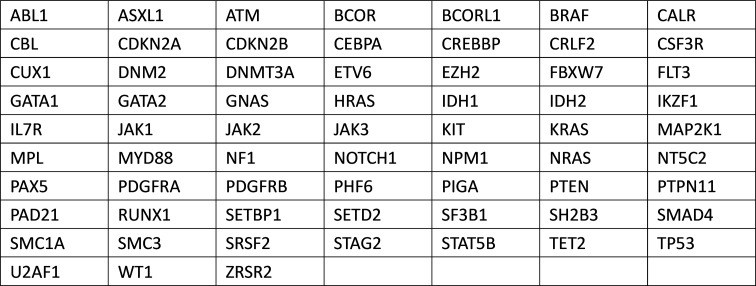
The detection range of NGS

### Classification of SM

The mutated genes DNMT3A, ASXL1, TP53, RUNX1, JAK2 and JAK3 were classified into Unfavorable group, and BCOR/BCORL1 and PIGA were classified into Favorable group according to previous literature [7]. In this study, Genes with only three or fewer times were counted as “Others” group.

### Statistical analysis

The efficacy was evaluated at 1 year, and the follow-up was defined as 36 months from the time of treatment. The data were analyzed using SPSS version 22.0 (IBM, Armonk, NY) and Prism version 8.0 (GraphPad Software, San Diego, CA). Continuous variables not conforming to a normal distribution were expressed as the median (range) and compared using the rank-sum test, and categorical variables were expressed as frequency and compared using the chi-square test. Survival was analyzed by Kaplan-Meier survival analysis. A log-rank test was applied to compare the survival curves. For survival data, univariate analysis and multivariate Cox analysis were performed to evaluate the risk factors for prognosis. Variables with a P value < 0.1 in univariate analysis were selected for the stepwise analysis and input into the multivariate Cox regression model. A P value < 0.05 was considered statistically significant.

## Results

### Patient characteristics

A total of 166 AA patients were divided into HSCT (128 cases, 77.1%) and IST (38 cases, 22.9%) groups according to treatment, the median age of the patients in the two groups was 29 years (9 ~ 66) and 43 years (15 ~ 75), respectively, and the difference was statistically significant (*p* < 0.001). The incidence of somatic mutations was 49.2% and 44.7%, respectively. In terms of efficacy response, the OR of HSCT group was 85.9%, which was significantly higher than IST group (*p* < 0.001), the overall survival was 85.9% and 81.6%, respectively, and the median follow-up was 14 (1 ~ 36) months and 18 (1 ~ 36) months, respectively. And the basic characteristics of the two groups are shown in Table 2.

**Table 2:** Association ananlysis of clinical characteristics with treatment in AA

Characteristic	*N*	HSCT	IST	*P*
N	166	128(77.1%)	38(22.9%)	
Sex-no, (%)				0.933
Male	84(50.6%)	65(50.8%)	19(50%)	
Female	82(49.4%)	63(49.2%)	19(50%)	
Age, year				**< 0.001**
Median	32	29	43	
Range	9 ~ 75	9 ~ 66	15 ~ 75	
SM, (%)	80(48.2%)	63(49.2%)	17(44.7%)	0.516
Response				**< 0.001**
CR	116(69.9%)	101(78.9%)	15(39.5%)	
PR	20(12.1%)	9(7.0%)	11(28.9%)	
NR	30(18.0%)	18(14.1%)	12(31.6%)	
Survival, (%)	141(84.9%)	110(85.9%)	31(81.6%)	0.512
Follow-up, M(range)	14(1 ~ 36)	14(1 ~ 36)	18(1 ~ 36)	0.258

Bold indicates that the P- value is statistically significant

### Distribution of mutated genes

**Fig. 1:**
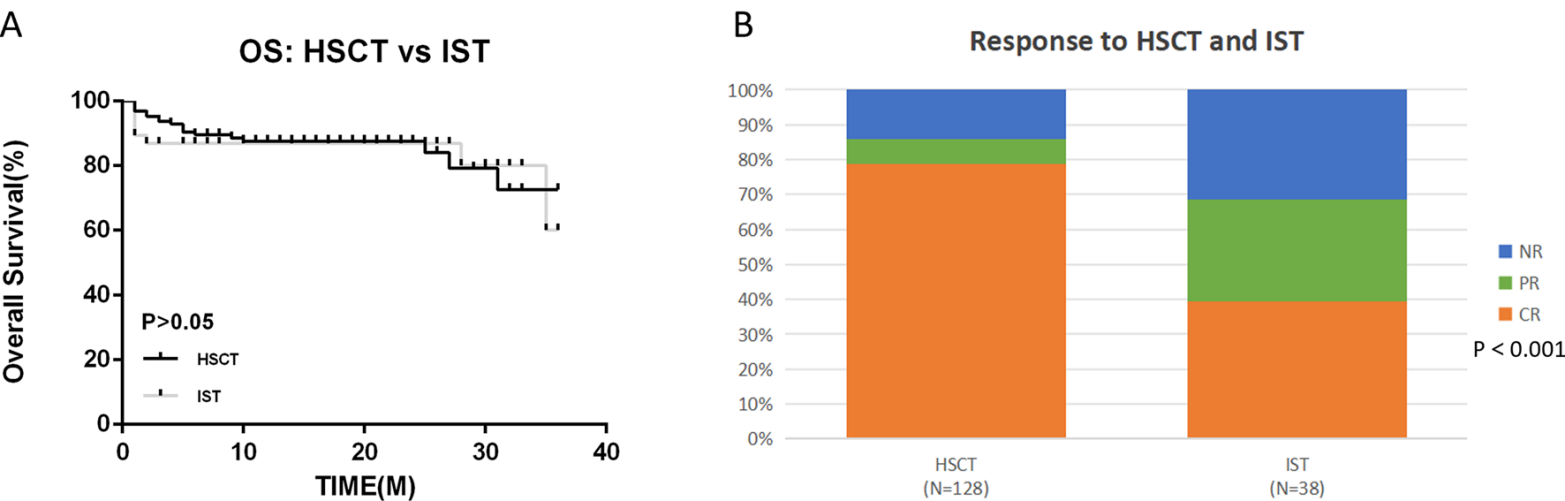
Survival outcomes and Response in the HSCT and IST groups. (**A**) OS. (**B**) Response

A total of 151 somatic mutations were detected in 84 (50.6%) patients, of which 42 (25.3%) had a single mutation and 26 (15.7%) had two mutations. And the 5 genes with the greatest number were BCOR/BCORL1 (12.6%), ASXL1 (8.6%), TET2 (6.6%), CEBPA (5.3%), and GATA2 (4.6%). The ranking of somatic mutations frequency is shown in Fig. 1.

### Mutations and prognosis

The 166 AA patients were divided into SM group (84, 50.6%) and No-SM group (82, 49.4%) according to mutation or not, and the median age in the two groups was 35 years (9 ~ 75) and 27 years (9 ~ 68), respectively, and they were relatively older when the mutation was detected(*p* = 0.01). The OR were 82.1% and 81.7%, and OS were 84.5% and 85.4%, respectively. Find details on somatic mutations, their effects and prognosis are shown in Table 3.

**Table 3:** Association ananlysis of clinical characteristics with presence or absence of somatic mutations in AA

Characteristic	Somatic mutation	No-somatic mutation	*P*
N	84(50.6%)	82(49.4%)	
Sex-no, (%)			0.642
Male	41(48.8%)	43(52.4%)	
Female	43(51.2%)	39(47.6%)	
Age, year			**0.01**
Median	35	27	
Range	9 ~ 75	9 ~ 68	
Treatment, (%)			0.516
HSCT	63(75%)	65(79.5%)	
IST	21(25%)	17(20.5%)	
Response			0.901
CR	60(71.4%)	56(68.3%)	
PR	9(10.7%)	11(13.4%)	
NR	15(17.9%)	15(18.3%)	
Survival, (%)	71(84.5%)	70(85.4%)	0.880

Bold indicates that the P- value is statistically significant

### Mutation type, treatment and prognosis

We divided all AA patients into four groups based on the type of SM: Favorable, Unmutated, Unfavorable, and Others. The results showed that patients with Favorable mutations had a significantly higher response compared to those with Unfavorable mutations (OR 93.7% vs. 70.8%, *p* < 0.05), however, there was no statistically significant differences between other groups. The information about the type of SM in all patients is shown in Table 4.

**Table 4:** Association ananlysis of clinical characteristics with different somatic mutations in AA

Characteristic	Favorable	Unmutated	Unfavorable	Others	
N	16	82	24	44	
Sex-no, (%)					
Male	7(43.7%)	43(52.4%)	12(50%)	21(48.8%)	
Female	9(56.3%)	39(47.6%)	12(50%)	22(51.2%)	
Age, year					
Median	35	27	35	35	
Range	20 ~ 75	9 ~ 68	19 ~ 75	9 ~ 66	
Treatment, (%)					
HSCT	13(81.2%)	65(79.3%)	19(79.2%)	31(70.5%)	
IST	3(18.8%)	17(20.7%)	5(20.8%)	13(29.5%)	
Response					
CR	13(81.2%)	56(68.3%)	15(62.5%)	32(72.7%)	
PR	2(12.5%)	11(13.4%)	2(8.3%)	5(11.4%)	
NR	1(6.3%)	15(18.3%)	7(29.2%)	7(15.9%)	
Survival, (%)	15(93.7%)	70(85.4%)	19(79.2%)	37(84.1%)	

We divided the HSCT and IST groups into four subgroups based on the type of SM, respectively. The results showed that the response and survival of patients with Favorable mutations in the HSCT group were better than those with other types of mutations. The results showed that the HSCT-Favorable group demonstrated a significantly better response and survival when compared to the IST-Favorable group(OR 100% vs. 67.7%, *p* < 0.05) (3-year OS 100% vs. 67.7%, *p* < 0.05). The information about the type of SM in HSCT and IST groups is shown in Table 5.

**Table 5:** Type of mutation in HSCT and IST groups

Characteristic	HSCT (*N* = 128)				IST (*N* = 38)			
	Favorable	Unmutated	Unfavorable	Others	Favorable	Unmutated	Unfavorable	Others
N	13	65	19	31	3	17	5	13
Response								
CR	13(100%)^A^	51(78.5%)	13(68.4%)^C^	24(77.4%)	0	5(29.4%)^E^	2(40%)	8(61.5%)
PR	0	6(9.2%)	1(5.3%)	2(6.5%)	2(67.7%)	5(29.4%)	1(20%)	3(23.1%)
NR	0	8(12.3%)	5(26.3%)	5(16.1%)	1(33.3%)	7(41.2%)	2(40%)	2(15.4%)
Survival(%)	13(100%)^B^	57(87.7%)	14(73.7%)^D^	26(83.9%)	2(67.7%)	13(76.5%)^F^	5(100%)	11(84.6%)

A: HSCT-Favorable vs. IST-Favorable (OR 100% vs. 67.7%, ***p*** **< 0.05**)

B: HSCT-Favorable vs. IST-Favorable (3-year OS 100% vs. 67.7%, ***p*** **< 0.05**)

C: HSCT-Favorable vs. HSCT-Unfavorable (OR 100% vs. 73.7%, *p* = 0.072)

D: HSCT-Favorable vs. HSCT-Unfavorable(3-year OS 100% vs. 73.7%, *p* = 0.072)

E: IST-Unmutated vs. IST-Others (OR 58.8% vs. 84.6%, ***p*** **= 0.018**)

F: IST-Unmutated vs. IST-Others (3-year OS 76.5% vs. 84.6%, *p* = 0.202)

※One patient in the mutation group combined BCOR + JAK3 mutation, two patients combined BCORL1 + DNMT3A mutation, one patient combined BCOR + ASXL1 mutation, one patient combined BCOR + PIGA + ASXL1 mutation, and one patient combined BCOR + DNMT3A + JAK2 mutation, all six cases were included in the Unfavorable group.

### Number of mutations and prognosis

We grouped all AA patients according to the number of SM, and found that when patients had three or more SMs, there was a noticeable decline in both efficacy and prognosis. The detailed data regarding the number of SMs can be found in Table 6.

**Table 6:** Number of somatic mutations and prognosis in AA

Characteristic	0	1	2	≥ 3
N	82	42	26	16
Response				
CR	56(68.3%)	30(71.4%)	20(76.9%)	10(62.5%)
PR	11(13.4%)	5(11.9%)	3(11.5%)	1(6.2%)
NR	15(18.3%)	7(16.7%)	3(11.6%)	5(31.3%)
Survival, (%)	70(85.4%)	36(85.7%)	23(88.5%)	12(75%)

### Univariate and multivariate analysis

Analysis of factors associated with survival and therapy response is detailed in Table 7. Univariate analysis identified that age greater than 40 years old (*P* = 0.021) and VSAA (*P* = 0.032) are risk factors that impact survival. Additionally, age greater than 40 years old (*P* = 0.010) was found to be a risk factor affecting the response. Multivariate analysis revealed that age greater than 40 years old (*P* = 0.026, OR = 2.465,95%CI: 1.114–5.455) and VSAA (*P* = 0.037, OR = 2.321,95%CI: 1.054–5.114) are independent risk factors for survival. Age greater than 40 years old (*P* = 0.013, OR = 2.509,95%CI: 1.211–5.196) emerged as an independent risk factor for the response.

**Table 7:** Analysis of factors associated with survival and therapy response.

Variable	OS		Response	
	Univariate, *P*	Multivariate, *P*(HR,95%CI)	Univariate, *P*	Multivariate, *P*(HR,95%CI)
Treatment, HSCT vs. IST	0.851	-	0.120	-
Age, ≤ 40 year vs. > 40yr	**0.021**	**0.026**,**(2.465**,**1.114–5.455)**	**0.010**	**0.013**,**(2.509**,**1.211–5.196)**
Sex, Male vs. Female	0.875	-	0.901	-
Diagnosis, NSAA + SAA vs. VSAA	**0.032**	**0.037**,**(2.321**,**1.054–5.114)**	**0.075**	**0.077**,**(1.921**,**0.932–3.958)**
Interval from diagnosis to HSCT, ≤ 1 year vs. > 1yr	0.780	-	0.503	
Somatic mutation, yes vs. no	0.938	-	0.638	-
Number of mutations, 0/1 vs. ≥ 2	0.857	-	0.925	-
Type of mutations, Favorable vs. Others	*p* > 0.05	-	*p* > 0.05	-

### Discussion and conclusion

**Fig. 2:**
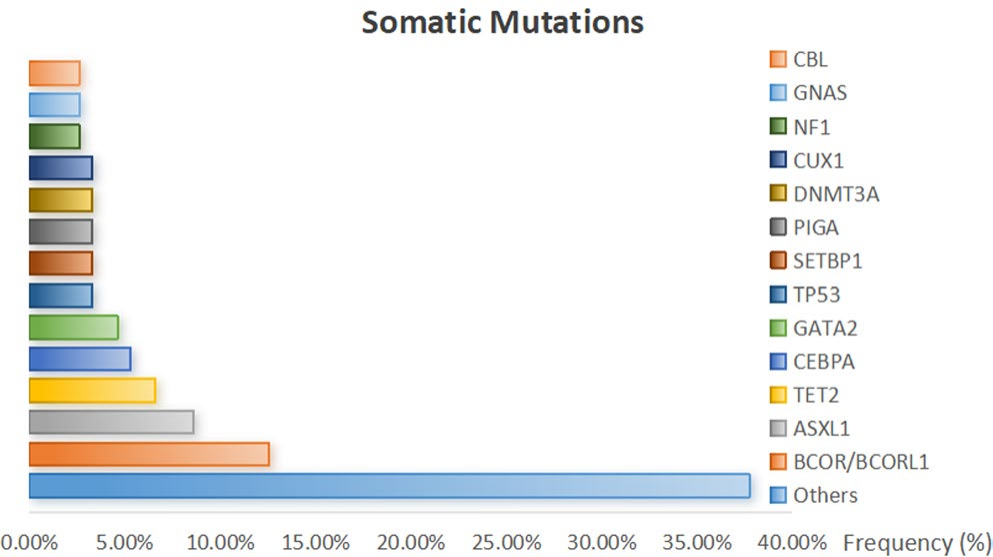
Somatic mutations frequency. Genes with only three or fewer times were counted as “Others”

Currently, HSCT and IST are the primary radical treatments for AA. However, several factors must be assessed before selecting a treatment plan, including the patient’s age, disease severity, availability of donors, and economic status [16]. Due to challenges such as treatment resistance, recurrence, and clonal evolution, the 10-year overall survival (OS) and failure-free survival (FFS) rates for first-line IST in AA patients are only 55% and 40%, respectively, which were significantly lower than those achieved with HSCT. Over the past decade, the therapeutic efficacy of HSCT has substantially improved, with long-term survival rates approaching 70–90%, making it the preferred treatment for AA patients with a suitable donor [5, 16]. In this study, patients who underwent HSCT as the initial treatment exhibited a higher response rate compared to those who received IST (OR 85.9% vs. 68.4%, *p* < 0.05). However, no significant difference was observed in long-term survival rates (3-year OS 85.9% vs. 81.6%, *p* > 0.05) (Fig. 2).

Previous research has revealed the regularity of SM in AA patients. Yoshizato’s studies have identified the most frequently mutated genes in AA patients as BCORL1, BCOR, ASXL1, DNMT3A, and TET2 [7, 8, 17]. Our findings align with these, with BCOR and BCORL1 being the most prevalent mutations, followed by the epigenetic-related mutations ASXL1 and TET2. Additionally, the transcription factor-related mutations GATA2 and CEBPA were also notably present in our study (Fig. 1). Some studies have revealed the relationship between these mutations and AA. For instance, BCOR plays a role in embryonic development, mesenchymal stem cell function, and the development of hematopoietic and lymphatic systems [18]. It is one of the most common mutations in AA patients and is associated with a positive response to IST. ASXL1 and DNMT3A mutations are common in both AA and AML/MDS. The DNMT3A mutation results in the accumulation of cells in the bone marrow due to impaired differentiation [19], while ASXL1 is crucial in the pathogenesis of leukemia by altering histone modifications and increasing the risk of transformation to MDS [20]. TET2, involved in DNA demethylation, regulates cell differentiation and proliferation, and some studies suggest that mutations in TET2 may reduce the risk of MDS transformation [21]. GATA2 regulates the transition from early embryonic endothelial to hematopoietic cells and promotes the formation of hematopoietic stem cells. GATA2 mutations can lead to a loss of NK cell progenitors, reduced NK cell differentiation, and immune dysfunction, which can manifest early in AA or progress to AA, indicating a role for GATA2 in AA pathogenesis [22–24]. Although CEBPA mutations are frequently observed in AML patients and are associated with sensitivity to chemotherapy [25], and a better prognosis compared to CEBPA wild-type AML patients [26], there are limited reports on CEBPA in AA. Further research is warranted to explore the relationship between CEBPA mutations and AA.

Another significant challenge in the treatment of AA patients, whether following HSCT or IST, is clonal evolution. Studies have indicated that the incidence of secondary myeloid tumors in AA patients after IST can be as high as 20% within 10 years [17, 18, 27–29]. The proportion of patients with unfavorable mutations, such as ASXL1, RUNX1, and SETBP1, who progress to MDS/AML after HSCT, is alarmingly high, reaching 40% after 7–8 years. This is 15-25% higher than in patients without these mutations [7, 8, 30, 31]. In contrast, the risk of progression to MDS/AML for mutations in BCOR and BCORL1 is significantly reduced (< 5%) [21, 29, 30, 32]. In our study, only three patients in the HSCT group developed Post-transplant Lymphoproliferative Disorder (PTLD), and one patient developed a secondary thyroid tumor, while one patient in the IST group progressed to AML after three years. It is important to note that our follow-up period was only three years, and thus, the incidence of clonal evolution, particularly in patients with mutations in ASXL1, DNMT3A, RUNX1, and TP53, which are more likely to convert to AML/MDS, requires close attention during follow-up. Additionally, CH increases the risk of cardiovascular diseases and hematological malignancies (HM), which is now considered a precancerous condition [10, 33–35]. Therefore, understanding the somatic mutations (SM) of patients through NGS is helpful to guide the treatment plan and predict prognosis.

**Fig. 3:**
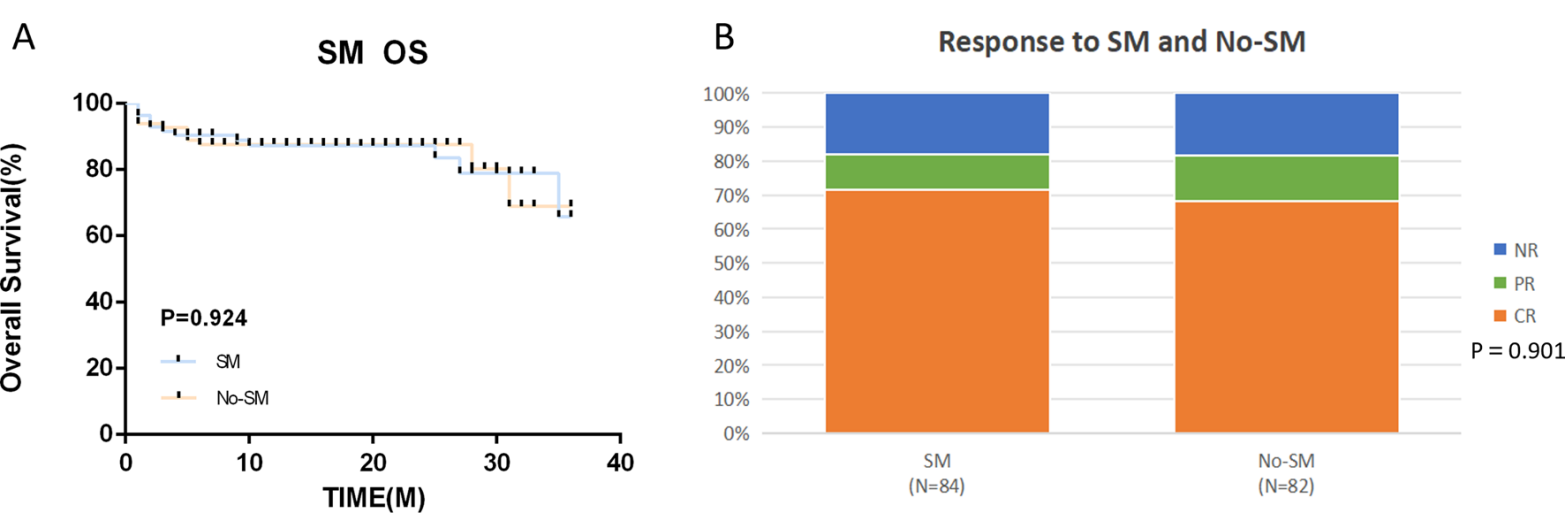
Survival outcomes and Response in the SM and No-SM groups. (**A**) OS. (**B**) Response

**Fig. 4:**
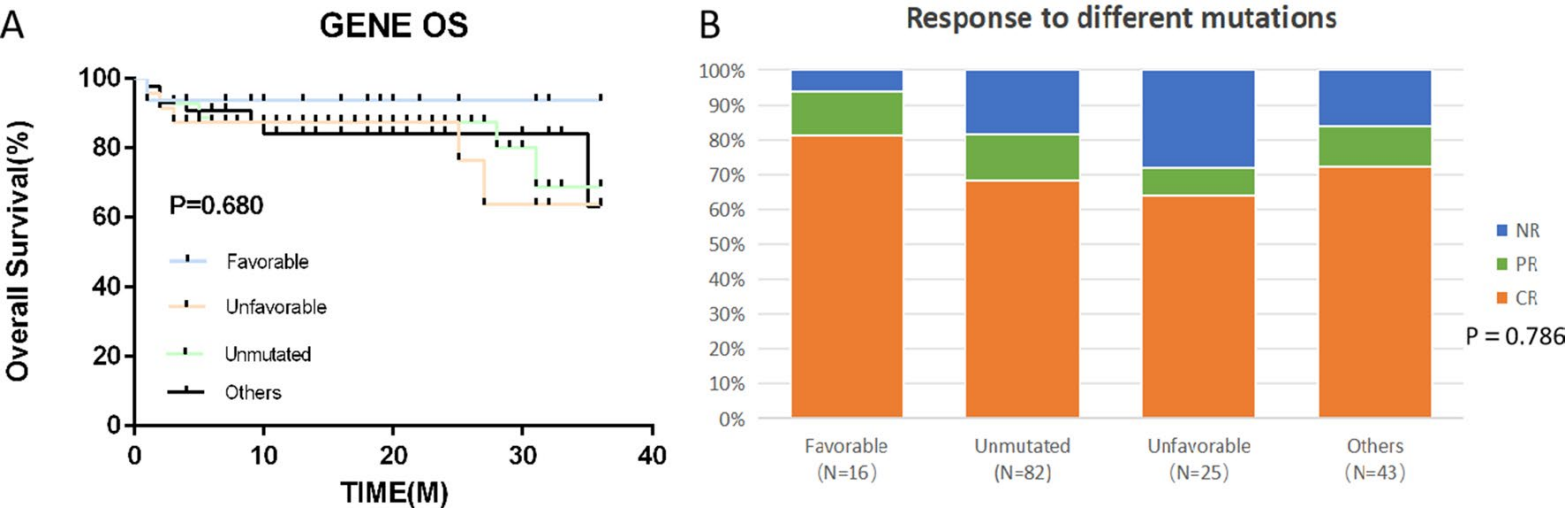
Survival outcomes and Response with different somatic mutation type. (**A**) OS. (**B**) Response

**Fig. 5:**
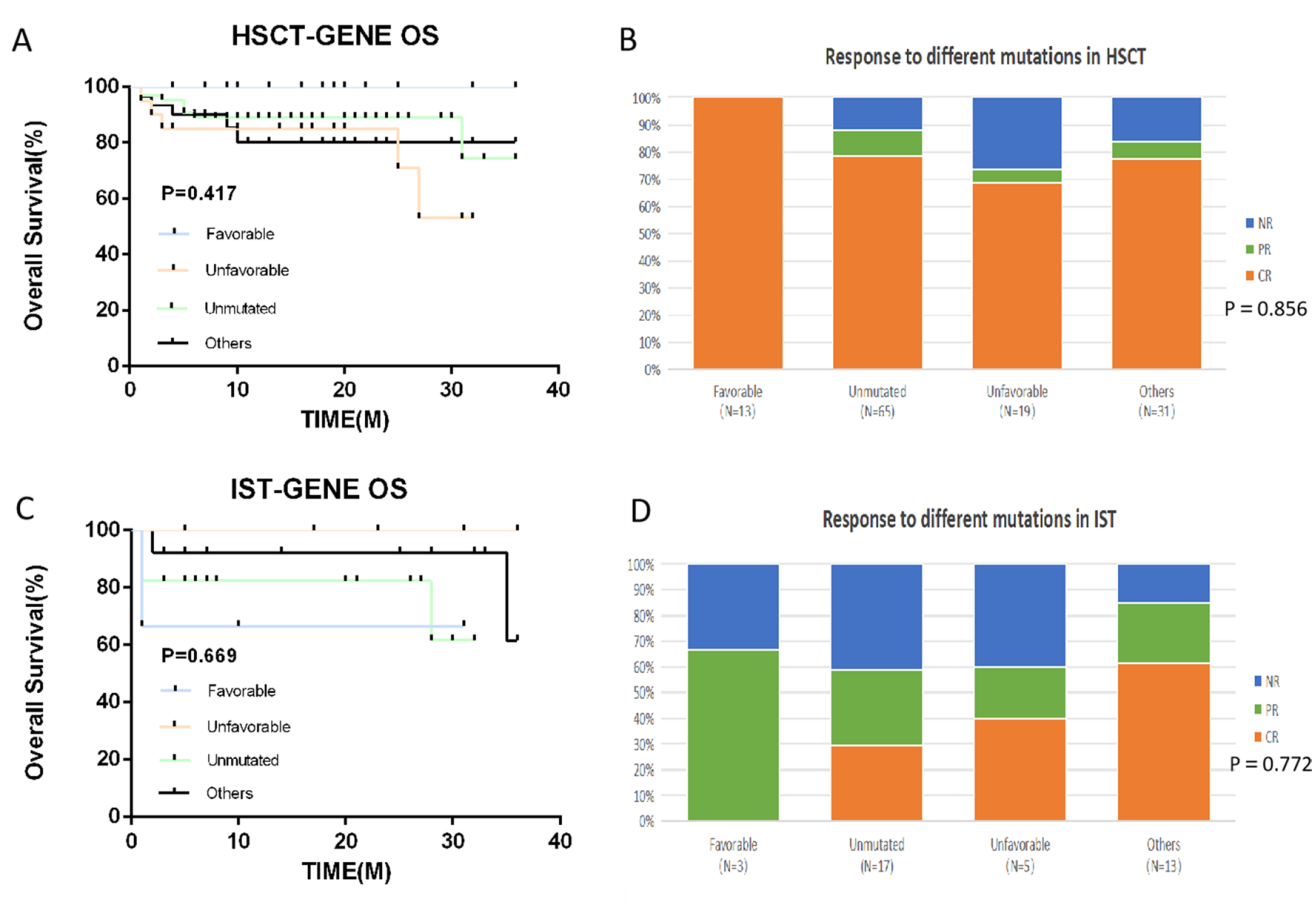
Survival outcomes and Response with different somatic mutation type in HSCT and IST groups. (**A**) HSCT-OS. (**B**) HSCT-Response. (**C**) IST-OS. (**B**) IST-Response

**Fig. 6:**
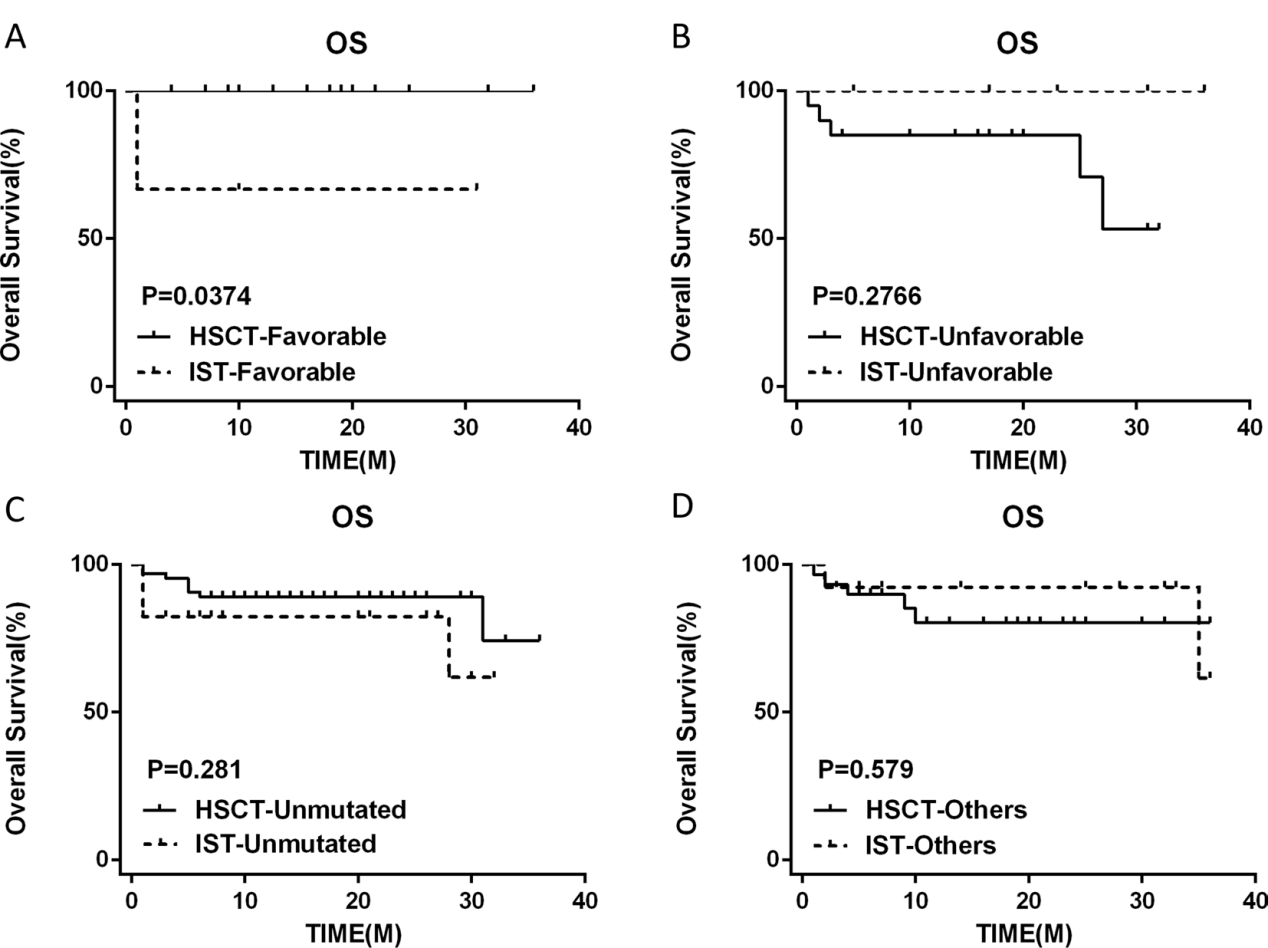
Survival outcomes with different somatic mutation type in different groups. (**A**) Favorable. (**B**) Unfavorable. (**C**) Unmutated. (**D**) Others

In this research, we categorized patients into two groups based on somatic mutation status: Somatic Mutation (SM) and No-SM. Our analysis revealed no significant disparities in treatment response (OR 82.1% vs. 81.7%, *p* > 0.05) and survival rates (OS 84.5% vs. 85.4%, *p* > 0.05), as illustrated in Fig. 3, aligning with the findings of Lingling Liu et al. [36] and Park et al. [9]. However, the impact of mutation type on treatment remains a central interest. We further stratified all patients into four distinct groups based on the type of mutation: Favorable, Unfavorable, Unmutated, and Others. Our findings indicated that the Favorable group exhibited a markedly superior response compared to the Unfavorable group (OR 93.7% vs. 70.8%, *p* < 0.05) and survival (3-year OS 93.7% vs. 79.2%, *p* < 0.05) (Fig. 4). Additionally, we subdivided the HSCT and IST groups into these four categories. Within the HSCT group, we observed a notably higher response in the subgroup with favorable mutations compared to those with unfavorable mutations, although this difference did not reach statistical significance (OR 100% vs. 73.7%, *p* = 0.072). Similarly, the 3-year OS also favored the favorable group (3-year OS 100% vs. 73.7%, *p* = 0.072). Interestingly, within the IST group, we dentified a significant trend where patients harboring other utations exhibited a higher response than those without mutations (OR 84.6% vs. 58.8%, *p* = 0.018) (Fig. 5). Notably, the HSCT-Favorable group demonstrated superior response and survival compared to the IST-Favorable group (OR 100% vs. 67.7%, *p* < 0.05) (3-year OS 100% vs. 67.7%, *p* < 0.05), while no significant differences were observed in the other subgroups (Fig. 6). These results suggest that in AA patients who harbor favorable mutations such as PIGA and BCOR/BCORL1, the efficacy and prognosis of first-line HSCT treatment are significantly improved. This underscores the importance of SM detection via NGS before treatment [37, 38]. For newly diagnosed AA patients with such favorable mutations and a suitable donor, Allogeneic-HSCT should be considered a priority. Interestingly, we also observed that within the IST group, patients with other mutations had a higher response compared to those without mutations (OR 84.6% vs. 58.8%, *p* = 0.018). This finding may suggest that partial mutations could serve as an adaptive mechanism to counteract the disease’s effects.

**Fig. 7:**
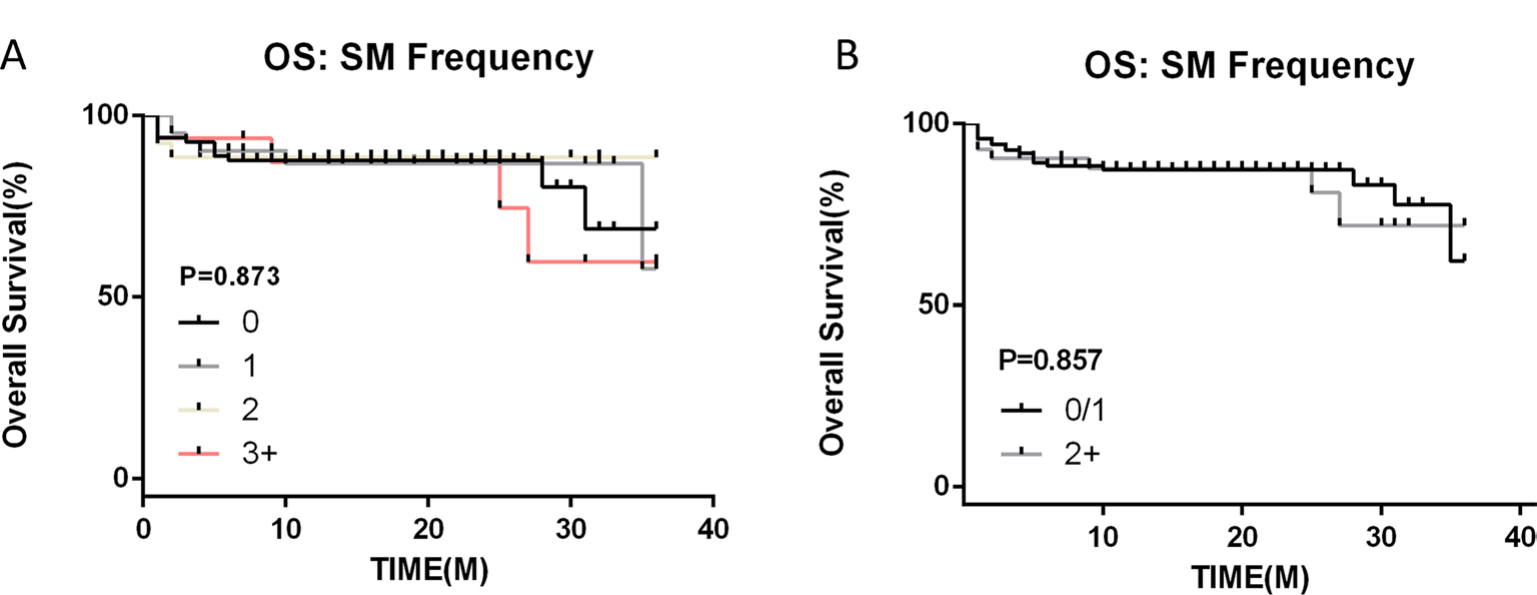
Survival outcomes with different somatic mutation number. (**A**) 0/1/2/3+. (**B**) 0,1/2+

Currently, there is a dearth of literature addressing how the quantity of SM in AA patients influences treatment efficacy and prognosis. In our study, we classified all patients based on the number of SM they harbored. Our analysis revealed that there was no significant difference in treatment efficacy and prognosis when patients had 0, 1, or 2 mutations. However, when the number of SM in AA patients increased to three or more, we observed a notable decline in both efficacy and prognosis (Fig. 7). This observation leads us to hypothesize that the total number of SM might exert an impact on the treatment outcomes and prognosis of AA patients. Furthermore, this potential influence could be intertwined with the specific types of mutations present in AA patients, as well as the treatment strategies employed. Given these preliminary findings, we advocate for additional research to elucidate the complex interplay between mutation count, mutation type, and treatment efficacy in AA patients. Such studies could provide valuable insights and guide the development of more personalized and effective treatment plans.

## Conclusion

This study revealed that the incidence of CH in AA patients is approximately 50.6%. The five genes with the highest mutation rates are BCOR/BCORL1 (12.6%), ASXL1 (8.6%), TET2 (6.6%), CEBPA (5.3%), and GATA2 (4.6%). Notably, AA patients with favorable mutations, such as BCOR/BCORL1, exhibit a higher response and better survival outcomes compared to those without mutations or those with unfavorable mutations such as ASXL1/DNMT3A. HSCT is recommended as the first-line treatment for AA patients with favorable mutations if a suitable donor is available. Conversely, AA patients with unfavorable mutations should undergo regular follow-up after IST if a donor is not available, to prevent the development of CH. However, our study acknowledges several limitations. Firstly, as a retrospective study, the sample size of the IST group was relatively small, leading to unbalanced data between groups and potentially less pronounced differences in subgroup analyses. Therefore, further well-designed, prospective, and controlled studies, as well as long-term prospective studies in AA patients, are necessary to elucidate the prognostic significance of these mutations.

## Electronic supplementary material

Below is the link to the electronic supplementary material.

Supplementary Material 1

## Data Availability

No datasets were generated or analysed during the current study.
