# Supplementary material for: Hematopoietic stem cell transplantation and immunosuppressive therapy: implications of clonal haematopoiesis
Source: Ann Hematol. 2025 Jan 28;104(3):1877–86. doi: 10.1007/s00277-024-06152-6 (PMC12031881; doi:10.1007/s00277-024-06152-6)
Supplement: Supplementary file 1 — Supplementary Material 1 [file 277_2024_6152_MOESM1_ESM.docx]

The results showed that Renal anemia was the main causes of PR and Covid-19 was the main causes of NR in HSCT group. Non-remission of hemogram was the main cause of PR and Persistent thrombocytopenia was the main cause of NR in the IST group.

| PR（N=20） | HSCT（N=9） | IST（N=11） |
| --- | --- | --- |
| Persistent thrombocytopenia | 2 | 6 |
| Renal anemia | 5 | 2 |
| TMA nephropathy | 1 | 0 |
| cGVHD | 1 | 0 |
| Non-remission of hemogram | 0 | 3 |

| NR（N=30） | HSCT（N=18） | IST（N=12） |
| --- | --- | --- |
| Covid-19 | 6 | 2 |
| Respiratory failure | 5 | 2 |
| Acute renal failure | 4 | 1 |
| Pulmonary hemorrhage | 0 | 1 |
| Intracerebral hemorrhage | 1 | 1 |
| TA-TMA | 1 | 0 |
| Deep coma | 1 | 0 |
| Non-remission of hemogram | 0 | 5 |

we directly compared the subgroups of the HSCT group with those of the IST group. The results are shown in the table below.

| HSCT-*P* (OR/OS) | Unfavorable | Unmutated | Others |
| --- | --- | --- | --- |
| Favorable | **0.072/0.072** | 0.221/0.184 | 0.121/0.121 |
| IST-*P* (OR/OS) | Unfavorable | Unmutated | Others |
| Favorable | 0.498/0.196 | 0.940/0.794 | 0.189/0.189 |

The six patients in question harbored a combination of favorable and unfavorable mutations, and their clinical information and efficacy are shown in the table below.

| Patient number | Somatic mutation | VAF | Treatment | therapeutic response |
| --- | --- | --- | --- | --- |
| 1 | BCORL | 47.8% | HSCT | CR |
|  | DNMT3A | 57.9% |  |  |
| 2 | BCOR | 2.4% | HSCT | CR |
|  | JAK3 | 47.6% |  |  |
| 3 | ASXL1 | 91.2% | HSCT | NR( COVID,Died ) |
|  | BCOR | 47.6% |  |  |
| 4 | ASXL1 | 78.3% | HSCT | CR |
|  | BCOR | 2.6% |  |  |
|  | BCOR | 6.7% |  |  |
|  | PIGA | 3.1% |  |  |
| 5 | DNMT3A | 4.5% | HSCT | PR( Thrombocytopenia ) |
|  | BCORL1 | 99.9% |  |  |
| 6 | FLT3 | 48.47% | IST | NR（Multi-organ failure, Died） |
|  | BCOR | 54.3% |  |  |

The clinical data and efficacy in patients with GATA2 mutations are shown in the table below.

| Patient number | Somatic mutation | VAF | Treatment | therapeutic response |
| --- | --- | --- | --- | --- |
| 1 | GATA2 | 2.03% | IST | PR（Thrombocytopenia） |
| 2 | GATA2 | 78% | HSCT | PR（Thrombocytopenia） |
| 3 | GATA2 | 5.3% | HSCT | CR |
|  | GATA2 | 3.86% |  |  |
|  | DNMT3A | 2.01% |  |  |
| 4 | GATA2 | 33.06% | HSCT | NR（COVID，Died） |
| 5 | GATA2 | 23.6% | HSCT | CR |
| 6 | GATA2 | 24.1% | HSCT | CR |
